# Singing Interventions in Pulmonary Rehabilitation: A Scoping Review

**DOI:** 10.3390/ijerph20021383

**Published:** 2023-01-12

**Authors:** Soo Ji Kim, Myung Sun Yeo, So Yeon Kim

**Affiliations:** 1 Music Therapy Education, Graduate School of Education, Ewha Womans University, Seoul 03760, Republic of Korea; 2 Arts Education and Therapy Institute, Ewha Womans University, Seoul 03760, Republic of Korea; 3 Department of Music Therapy, Graduate School, Ewha Woman’s University, Seoul 03760, Republic of Korea

**Keywords:** singing, pulmonary rehabilitation, intervention, scoping review

## Abstract

(1) Background: Individuals with pulmonary disease need intensive and consistent rehabilitation due to their high risk for serious illness and long-term complications. The purpose of this scoping review was to provide a comprehensive analysis of relevant research regarding the use of singing in pulmonary rehabilitation. (2) Methods: A systematic literature search was performed using the PsycINFO, CINAHL, PubMed, and Web of Science databases. A search for studies that employed singing in pulmonary rehabilitation for patients with pulmonary disease was conducted. (3) Results: Studies that met the selection criteria were summarized and analyzed. Twenty-seven studies were included in the final analysis. Results showed that research using singing in pulmonary rehabilitation generally employed an intervention with structured tasks and additional home practice or socialization time. However, the singing procedure in each intervention was not always specifically described and the findings were inconsistent. (4) Conclusions: Programmed singing interventions can support lung health and be an effective component of pulmonary rehabilitation. The therapeutic singing method in relation to respiratory exercises should be integrated into the main activity in the intervention. Overall, singing has physical and psychosocial effects, leading to improvements in symptoms, but more research is necessary to ensure that the respiratory needs of people with pulmonary disease are adequately met.

## 1. Introduction

Breathing is a fundamental body activity that impacts every area of human function including communication and emotional state [1]. Since difficulty breathing causes problems in multiple areas of life, the scope of pulmonary rehabilitation research is expanding from improving specific respiratory function to supporting the psychosocial needs of patients with pulmonary disease (i.e., asthma, chronic obstructive pulmonary disease [COPD], lung cancer, pneumonia, and pulmonary fibrosis) [2]. Moreover, those with compromised lung functioning are at greater risk for serious illness, long-term complications, and mortality from COVID-19 [3,4]. Thus, the importance of pulmonary rehabilitation has never been greater, and new treatment modalities including home-based interventions and telerehabilitation are needed [5].

In the music therapy literature, diverse pulmonary rehabilitation programs have been implemented that applied various musical activities. These musical activities for respiratory rehabilitation included vocalization, singing, and diaphragmatic breathing, induced relaxation during music listening, and wind instrument playing [1,6,7]. Among these musical activities, singing has repeatedly been reported as an effective means of reducing the respiratory patients’ symptoms such as breathlessness and enhancing their quality of life [8,9].

Singing requires controlled voice production with controlled respiration, and physical, psychological, and social benefits have been reported from group and solo singing [8,10,11]. In general, singing involves holding the correct posture, executing a short, deep inspiration while contracting the diaphragm, and maintaining prolonged expiration. To achieve therapeutic goals in relation to respiratory function, musical elements should be modified to control target muscle movements during singing. Singing requires higher and more sustained expiratory pressure than speech to maintain voice loudness at a relatively constant level [12]. Singing actively involves the respiratory system, which drives the air pressure required to initiate and maintain vocal fold function as well as control vocal intensity [13]. Musical elements such as melody and dynamics can be used to influence the timing and strength of contractions of the respiratory muscles. As a result, singing has been demonstrated to improve respiratory function in various target groups such as patients with Parkinson’s disease, those with cerebral palsy, and patients with respiratory conditions such as asthma and COPD [14]. 

While positive outcomes have been reported in pulmonary rehabilitation research employing singing, small effect sizes have been found due to methodological challenges [15]. Researchers in this area have called for studies of longer duration and with larger sample sizes [16] and point to the significant role that singing could play in pulmonary rehabilitation due to its enjoyable nature, low risk, and minimal-to-no cost [14]. 

To encourage the use of singing as a therapeutic medium for pulmonary rehabilitation, it is first necessary to analyze the applications of singing for various pulmonary diseases. Hence, there is a need to map, organize, and synthesize the literature in this area, describe the range of interventions, and analyze their content and characteristics. Therefore, the aim of this study was to synthesize the available information regarding singing to promote pulmonary function in pulmonary rehabilitation. 

## 2. Methods

### 2.1. Scoping Review

A scoping review is a useful means of synthesizing research evidence and is often used to categorize literature in a specific field. It can be used to map the literature and clarify the boundaries and limitations of current interventions or to identify gaps in the research and make recommendations to address these gaps [17]. This scoping review employed Arksey and O’Malley’s (2005) five-stage framework. The findings from this scoping review are expected to inform the development of future singing protocols for respiratory rehabilitation in music therapy. Our focus of analysis included the structure and contents of singing interventions used to enhance the physiological and psychological health of people with pulmonary disease. 

### 2.2. Identifying Relevant Studies

In August and September of 2022, peer-reviewed journal articles were collected primarily through a search of four databases: PsycInfo, CINAHL, PubMed, and Web of Science. The main keywords and other diagnostic terms used in the search process were “respiration”, “pulmonary”, “asthma”, “emphysema”, “breathing”, and “COPD”, and the terminology related to musical techniques included “music”, “singing”, “choir”, and “voicing.” Other keywords such as “intervention”, “therapy”, “program”, “treatment”, “rehabilitation”, and “training” were also used. We also searched for target publications using the MeSH. The search strategy included terms related to pulmonary disease and music joined by a Boolean operator. Full-text publications that met the inclusion criteria and were published between 2002 and 2022 were identified. To avoid omitting research in the search process, the researchers changed the order of the search terms and repeated the search process on different dates.

### 2.3. Study Selection 

**Figure 1:**
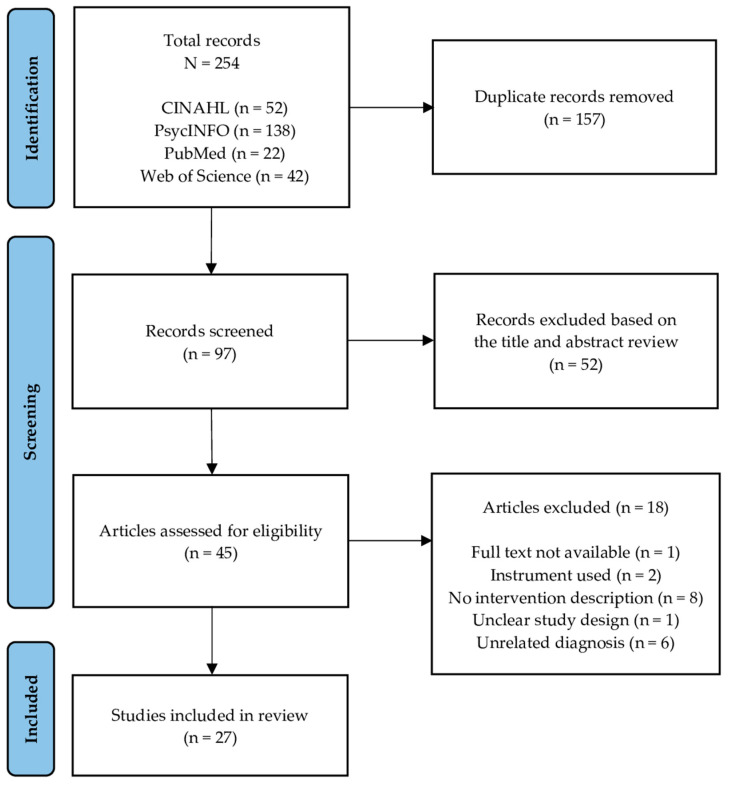
PRISMA flowchart describing the selection process of the included articles.

Published studies that used singing as an intervention for patients diagnosed with pulmonary disease were analyzed. Any type of intervention was acceptable as long as it utilized singing and aimed to treat a respiratory condition. Studies employing instruments or those in which singing was not the primary intervention were excluded. The delivery mode of the intervention could be individual or group sessions in a hospital ward or outpatient setting and could have been implemented for any length of time. Outcomes related to each intervention’s effectiveness in treating respiratory symptoms or psychosocial parameters (e.g., quality of life, anxiety) were all included. The search produced 254 results. Following the removal of 157 duplicate studies, the titles and abstracts of the remaining studies were reviewed, and an additional 52 studies were eliminated. The remaining 45 articles were read in full, and an additional 18 studies were found to not meet the study criteria and were also excluded. In the end, 27 studies were identified as meeting our inclusion criteria and were included in our analysis and review (see Figure 1). 

### 2.4. Data Charting Process

Data charting was performed in duplicate using a team approach. Results were compared following the initial data extraction, and charting forms were adjusted as needed. Studies were grouped based on outcomes relevant to the study objectives. Each article’s study design, intervention environment, participant information, treatment other than the music intervention, functional evaluation tools, and evaluation results were analyzed. The number of sessions per week, total sessions, and music intervention activities were also analyzed. For the intervention analysis, we used the original text to deliver the authors’ intended meaning.

## 3. Results

Twenty-seven studies were included in this review. Each study was analyzed, focusing on the research methods, type of intervention, characteristics of the participants, and research outcomes. Because this scoping review aimed to investigate the contents of singing interventions, intervention steps, tasks, and activities were also analyzed. All articles were published between 2002 and 2022, the period we specified for inclusion in this review. Three studies were published between 2002 and 2009, 13 studies were published between 2010 and 2019, and 11 studies were published in the last 3 years. The general characteristics of the analyzed studies are presented in Appendix A. More specific information about the studies’ interventions, outcomes, and findings are reported separately in Table 1.

### 3.1. Methodological Approaches

The most frequently conducted research method was randomized controlled trials (RCT) (n = 12) including one feasibility study [18] and two pilot studies [19,20]. Aside from RCT, there were five one-group pre-post design studies [21,22,23,24,25], three case studies [26,27,28] and descriptive studies including study protocols [29,30], and program evaluation [31,32]. Three qualitative studies were also found [33,34,35].

Singing interventions were mainly applied to the COPD patients (n = 18), and the other studies included mixed diagnoses (i.e., emphysema and COPD or cardiomyopathy from acquired heart disease and complex congenital heart disease). Except for two studies in which the participants’ mean age was 9 years (i.e., Wade, 2002) and 11.6 years (i.e., Irons et al., 2012), all studies included adults whose ages ranged from 25 to 72 years. In terms of outcome measures, we found four categories: lung function (i.e., peak expiratory flow rates or maximal expiratory pressure), psychological outcomes (i.e., satisfaction or depression), quality of life, and physical health (e.g., incremental shuttle walk test or 6 min walk test). Four studies indicated that their data were acquired from published articles using quantitative research methods and were secondary analyses of primary research. All studies explored the participants’ singing experiences and behavioral or psychological changes following the singing intervention. Three qualitative studies were conducted after quantitative studies were performed using objective measurements. One study [35] analyzed the participants’ satisfaction with the programs and perceived benefits to their physical and psychological well-being using content analysis of the data from [36]. Another study explored the experiences of COPD and chronic lung disease patients in a community-based group singing program [25]. This study used the same sample from the authors’ earlier study [33]. The third secondary analysis was carried out using a phenomenological approach to explore the inner changes of COPD patients through group singing, which was quantitatively analyzed by Clift et al. (2022) study.

### 3.2. Established Interventions vs. Researcher-Designed Interventions

Overall, two types of interventions were found: established interventions (n = 18) and researcher-designed interventions (n = 9). The established interventions in this review were community-based group singing programs supported by nonprofit foundations. One such intervention was Singing for Lung Health (SLH), in which professional singing teachers implemented the program to exercise participants’ physical, vocal, and breathing functions. Six studies used SLH [18,20,31,36,37,38] with the COPD patients. Another established intervention was SingStrong, which involved a symposium activity [21]. This intervention is similar to SLH, since both use a physical warmup, breathing, vocal exercises, and singing. However, SingStrong is focused on the participants’ personal objectives and subjective health and well-being. Other established interventions are Singing for Better Breathing [29,32,34,35,36,39,40], Sing Your Lungs Out [25,33], SINFONIA (**S**in**IN**g **F**or breathing in C**O**PD a**N**d **I**LD p**A**tients) [30], and HeartChoir [41].

The above studies were conducted to assess the effectiveness and applicability of specific singing interventions for individuals with pulmonary disease. All programs were established based on the benefits of group singing for subjective and objective health and well-being and targeted individuals with pulmonary disease including asthma and COPD. Two factors, socialization and home practice, were employed in most of the studies for maintaining and strengthening the participants’ engagement in the singing activity.

Regarding researcher-designed interventions, the singing application was systematically constructed with specific tasks to achieve functional goals. Similar to the established interventions, these included two to five steps of sub-components. Five interventions in this category employed a step-wise procedure with a home task, and three interventions had no home practice.

### 3.3. Expansion and Extension of Research on Singing Interventions

After arranging the collected studies in chronological order, some patterns emerged. Studies published between 2002 and 2009 [26,28,42] mainly reported the benefits of singing on lung function. Those published between 2010 and 2019 reported on interventions that were organized in the form of programs, and these systematic interventions were tailored to meet the needs of pulmonary rehabilitation. All of these interventions included two tasks: explicit breathing instruction and singing. Explicit breathing instruction included pursed-lip breathing, diaphragmatic breathing, and timed breathing with slow exhalation. These factors commonly appear in research on singing interventions for pulmonary rehabilitation. In addition, an attempt was made to focus on specific diagnoses including COPD. Most of the studies during this period were conducted with research funds. Intervention steps such as posture, diaphragmatic breathing, and respiratory exercise showed subdivided characteristics, confirming that various attempts were made to develop systematic programs. Only one of these 13 studies addressed the cross-cultural adaptation of the program [29] and considered the cultural relevance of the song selections and participation experience. Studies from 2020 onward were characterized by using the names of specific programs such as Singing for Lung Health [37,38,43], SingStrong [21,22,23], and Singing for Breathing [34,36]. These studies showed some variation that reflected the environmental challenges of conducting research during the COVID-19 pandemic. For example, SingStrong was applied in three studies with different steps: two steps (vocalization exercise and singing) and four steps (physical warmup, breathing, vocalization exercise, and singing). In response to the COVID-19 outbreak, the mode of session delivery was expanded to online programs or self-practice with supplementary materials at home in studies published since 2020.

### 3.4. Task Analysis of Singing Interventions

The focus of this scoping review was to analyze singing as a pulmonary rehabilitation intervention. Twenty one out of the 27 identified studies reported outcome measures related only to physical function. The steps in each intervention, categorized by pre-singing activity, singing, and post-singing activity, are presented in Table 1.

The major categories were identified in pre-singing activities: physical warmup including postural work; relaxation; and breathing or vocal exercises to facilitate diaphragmatic muscle movement. These intervention components were used in all studies except for Wade (2002). Postural work was primarily used to induce respiratory muscle exercises in advance of singing and included neck and head stretching, upper abdominal muscle stretching, or body percussion. Relaxation involved progressive muscle relaxation techniques or mindfulness exercises. The pre-singing breathing activities included pursed-lip breathing, diaphragmatic breathing, and timed breathing with slow exhalation. Some studies have provided instruction to participants prior to the start of the intervention, and this usually entailed breathing techniques to manage breathlessness [37]. Vocalization as a pre-singing activity involved vocalizing vowel sounds accompanied by musical elements. 

Some studies have offered instruction to enhance safety and better engage participants in the intervention. In eight studies, the researchers met with their participants to discuss song repertoire, provide health-related education, or offer instruction on breathing control techniques for enhancing self-management skills during singing [21,22,23,32,35,39,40,43]. In fact, all of the studies that performed SingStrong [21,22,23] included this pre-instruction discussion session, and this seems to have been helpful in situating and motivating participants. However, even in studies that utilized the same program, the pre-instruction steps varied.

**Figure 2:**
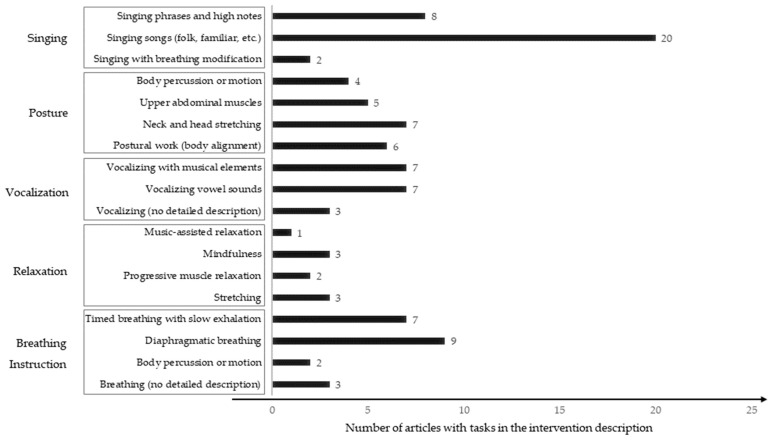
Singing-related activities described in the intervention procedure.

Unfortunately, descriptions about how each intervention designed or selected its singing activities are lacking in comparison to their descriptions of their pre-singing activities. Some studies have described song repertoire [22,41,42], while others have mentioned modifying musical elements in singing [17,18,24,29,37]. When we analyzed the singing-related activities in the interventions, we found that they fell into the following categories: singing, posture, vocalization, relaxation, and breathing instruction. All intervention tasks were described in conjunction with target respiratory functions, except singing (see Figure 2), which was described in terms of the song contents rather than the target respiratory functions. To be a therapeutic intervention in pulmonary rehabilitation, task-oriented, rationale-based intervention development is needed.

Home practice was analyzed as a post-singing activity in the interventions. In 17 studies, participants performed an additional task as home-based practice. This reflects the importance of regular singing in daily activities for individuals with pulmonary disease. To maintain the effects of the intervention in daily life, researchers encouraged participants to complete take-home assignments including homework and logs. Most homework included CDs or videos with simple instructions and music to sustain the activity.

Another interesting finding was that many studies described social activities after their intervention. Since their programmed intervention was intended to facilitate community activity, two studies encompassed event or group singing performances after intervention completion [21,35]. Other studies also emphasized socialization with online breakout rooms [22], workshops [39], and socialization activities [18,25,30,35] as post-singing activities.

### 3.5. Findings

Most quantitative studies reported on an improvement in the participants in physical or psychosocial areas. Seven studies reported positive changes in both domains [17,18,21,23,26,40,41], and one study reported no change in either domain [16]. Among the studies that measured physical function, 16 out of the 17 reported positive changes in this area. However, not all of the physical domains measured in these studies showed significant changes, and the parameters that showed changes were diverse, so there was no consistent trend observed. In addition, eight studies reported no change in physical function [16,22,24,25,35,37,38,39].

**Table 1:** Intervention characteristics, outcomes, and findings.

Author (Year)	Instructor, Mode, Frequency, Duration)	Structured Steps (Time Allocation)			Outcomes and Findings	
		Pre-Singing Activity (Time)	Singing (Time)	Post-Singing Activity/ Supplemental Activity	Physical Aspects	Psychosocial Aspects
Wade (2002) [28]	MT/Gr/15 min twice a week, 4 weeks	NR	Series of vocal exercises and singing (15 min)	NR	Increased or maintained lung function; PEFR after singing	Relaxation conditions were not consistent
Engen (2005) [26]	MT/Gr/45 min twice a week, 6 weeks	Brief warmup exercises involving body posture, breath control and support, phonatory exercises, vocalization	Progressively increased difficulty and songs with musical phrases to extend breath control	Break time (10 min) for socialization	Significant differences in breath management (extent of counting), breath support (intensity of speech); No significant differences in FEV, inspiratory threshold, distance walked, DUKE Health Profile physical health subscale	NA
Bonilha et al. (2009) [42]	Singing teacher, Physiotherapist/Gr/60 min once a week, 24 weeks	Physical relaxation (5 min), respiratory exercises with various breathing management (10 min) and vocalization exercises (15 min)	Singing training in Brazilian folk songs (30 min)	Practicing folk songs at home	Significant difference in MEP between groups	Significant improvements in QoL for both groups
Lord et al. (2010) [39]	Singing teacher/ Gr/60 min twice a week, 6 weeks	Postural work and physical stretches (10–20 min), breath observation and management (10 min), and vocal exercises (10 min)	Singing songs with (1) long phrases and (2) pauses more akin to natural speech (10–20 min)	Pre-session for learning breathing techniques “Help yourself” booklet for home practice	Significant differences in physical component score of SF36 and HADS between groups; No changes in ISWT, single breath counting, breath hold time	NA
Irons et al. (2012) [20]	MT/In/30 min daily, 8 weeks	Posture, diaphragmatic breathing, and vocal warmup (NR)	Singing songs containing high notes and sustained phrases (NR)	Encouraging singing diary	Significant differences in MIP and MEP	Improvements in some domains of the QoL for both groups
Lord et al. (2012) [40]	Singing teacher/Gr/60 min twice a week, 8 weeks	Posture, relaxation, and vocal exercises (NR)	Singing for breathing program (NR)	Homework and an accompanying CD of songs to practice at home	Significant difference in physical component score of SF36; No significant differences in breathing control measures, exercise capacity, daily physical activity	No significant differences in mental component score of SF36
Goodridge et al. (2013) [18]	MT/Gr/60 min once a week, 8 weeks	Relaxation and stretching exercises of the neck and arms (5 min); singing-related respiratory exercises, including incremental breathing (10 min), and vocalization exercises using ‘‘le,’’ ‘‘la,’’ ‘‘mi,’’ & ‘‘mu’’ sounds to sing selected songs (15 min)	Singing songs (30 min)	Practice singing at home on 2 other days that week (at least 15 min)	No significant difference in 6MWT	No significant differences in health-related QoL, SGRQ, BIPQ
Grasch et al. (2013) [24]	Home practice/In/10 min daily, 12 weeks	Series of breathing and vocal warmup (NR)	Singing a song of their choice (NR)	Singing each morning and evening guided by a singing pamphlet (5 min)	Significant differences in the Borg fatigue rating; No significant differences in FEV1, FVC, FEV1%, inspiratory and expiratory pressure	No significant differences in SGRQ, BSI-18
Pacheco et al. (2014) [27]	Singing teacher, physiotherapist/ Gr/60 min once a week, 10 weeks	Relaxation exercises of neck and upper and lower limb muscles (10–15 min), vocalization exercises (15 min)	Singing training using popular Portuguese songs (35 min)	Practice the songs at home during the week	No differences in residual volume and total lung capacity; three participants showed improvement in MEP after singing	No major differences in QoL
Gick & Daugherty (2015) [19]	Facilitators/ Gr/90 min once a week, 36 weeks	11 exercises, diaphragmatic breathing with a booklet (NR)	Group singing with lyrics using a karaoke machine. Familiar songs with various ranges. Reminded to apply breathing skills while singing (NR)	Home practice using the booklet and CD	Significant improvements in PEFR, vitality, and symptoms	Significant improvements in impact respiratory QoL; Significant decrease in negative mood, distress, and breathlessness in all groups
Mc- Naughton et al. (2017) [25]	Amateur singing group facilitator Gr/60 min once a week, 1 year	Sing Your Lungs Out— Warmup (5 min)	Singing of various genres (35 min)	Home practice with recordings of songs (optional)	Improvements in the 6MWT	Significant reduction in HADS
Trivedi (2017) [44]	Singing teacher/Gr/60 min twice a week, 4 weeks	Local relaxation of respiratory muscles (5 min), respiration practice as singing-related exercises, vocalization exercises by the loud/rhythmic pronunciation of vowels (5–10 min), demonstration of song and explanation of singing technique (5 min)	Singing practice (45 min) with intermittent rest (5 min) using Indian classical songs	NR	Significant improvements in dyspnea (MRC)	Significant improvements in QoL
Lewis et al. (2018) [31]	Singing facilitator/Gr/ 60–90 min once a week, 12 weeks	(SLH) physical warmups to correct posture and exercise respiratory muscles and the exhale for obstructive conditions. Vocalization with fricatives from a passive to a voiced exhale. Call and response sounds using different vocal qualities, range, dynamics, timbre, pitch, and rhythm	Rhythm and pitch games with a variety of melodic patterns to control the length of exhalation	Home practice with a repertoire to fit phrase lengths	Significant improvements in CAT	No significant improvements in general QoL, anxiety, patient activation, breathlessness, inhaler use
Ganzoni et al. (2020) [41]	Professional soprano singer/Gr/90 min once a week, 12 weeks	HeartChoir— NR	Singing lessons in a choir	Additional instructions for breathing exercises at home (20 min daily)	Significant differences in MIP; No significant differences in MEP, MVO2	Significant differences in QoL
Liu et al. (2019) [43]	Community nurse, MT/Gr/60 min once a week, 24 weeks	Relaxation exercises for neck and upper abdominal muscles (5 min), respiratory exercises (i.e., pursed lip breathing and diaphragmatic breathing (10 min), vocalization exercises including whispered hum or loud hum (15 min)	Singing exercise, participant-chosen music (30 min)	Home practice with copied songs on MP5 files (30 min daily)	NA	Significantly improved HADS, CCQ
Philip et al. (2020) [38]	Singing teacher/ Gr (face to face and online)/60 min once a week, 12 weeks	Singing for Lung Health— Physical warmup, breathing exercises, vocal warmup	Singing and a cool down	Encouraged daily practice using CD	NA	Improvements in PHQ-9. ABC Scale
Cahalan, Green et al. (2022a) [21]	Singing teacher Gr/60 min once a week, 8 weeks	SingStrong— Physical warmup and breathing exercises (15 min)	Singing (30 min)	Home practice with lyric book and CD, SingStrong symposium performance	Statistically significant improvement in 6MWT; No significant improvements in CAT	No significant improvements and nonsignificant worsening in HADS; Positive qualitative feedback, including improvements in breathing, QoL, intervention enjoyment
Cahalan, Meade et al. (2022b) [22]	Choir leader, vocal coach/ Gr (online)/45 min twice a week, 10 weeks	SingStrong—online Mindfulness, body scan, and gentle seated warmup (15 min), breathing retraining, vocal exercises (15 min)	Group singing (15 min)	Optional online breakout rooms for socialization	Improvements in all breathlessness symptoms, fatigue, usual activities, pain/disability, voice quality, communication/cognition	Improvements in breathing, general well-being (positive qualitative feedback)
Cahalan, Russell et al. (2022c) [23]	Respiratory physiotherapist, vocal coach/Gr (online)/45 min once a week, 12 weeks	SingStrong—online Brief physical warmup, vocal exercise, breathing to encourage diaphragmatic and intercostal muscle recruitment (20 min)	Singing (20 min) participant-chosen songs, informed on disease-related limitations of breathlessness and work of breathing	Session recording available for home practice	NA	Significant differences in IPF-PROM, QoL; Strong satisfaction with classes and improved efficacy
Clift et al. (2022) [36]	Facilitators/ Gr/90 min once a week, 10 weeks	Singing for Lung Health— Breathing exercise	Singing	Self-practice was encouraged using video resource (Price & Skingley, 2022)	NA	No significant differences except the physical role sub-scale on SF-36
Kaasgaard et al. (2022) [37]	Singing teacher/Gr/90 min twice a week, 10 weeks	Singing for Lung Health— Physical warmup (20 min), vocal & breathing exercises focusing on respiratory muscle movements (20 min)	Singing (40 min)	NR	Significant improvement on 6MWD; No significant between-group differences in lung function, dyspnea, adherence	Significant improvement on SGRQ; No significant between-group differences concerning SGRQ, HADS

Notes. ABC Scale = Activity Specific Balance Confidence Scale; BIPQ = Brief Illness Perceptions Questionnaire; BSI-18 = Brief Symptom Inventory-18; CAT = COPD Assessment Test; CCQ = Clinical COPD Questionnaire; FEV = forced expiratory volume; FEV1 = forced expiratory volume in 1 s; FEV1% = forced expiratory volume 1%; FVC = forced vital capacity; Gr = group session; HADS = Hospital Anxiety and Depression Scale; In = individual session; IPF-PROM = Idiopathic Pulmonary Fibrosis-Patient Reported Outcome Measures; ISWT = Incremental Shuttle Walk Test; MEP = Maximal Expiratory Pressure; MIP = Maximal Inspiratory Pressure; MRC = Medical Research Council Dyspnea Scale; MVO2 = maximum oxygen uptake during exercise; NA = not applicable; NR = not reported; MT = music therapist; PEFR = peak expiratory flow rate; PHQ-9 = Patient Health Questionnaire; QoL= quality of life; SF36 = Short Form 36; SGRQ = St. George’s Respiratory Questionnaire; SLH = Sing for Lung Health; 6MWD = 6 Minute Walking Test Distance; 6MWT = 6 Minute Walking Test.

## 4. Discussion

In this scoping review, we analyzed the research on singing in pulmonary rehabilitation. We examined the following elements of the studies: intervention type and characteristics and the participants’ characteristics. The following main findings emerged from our review.

First, considering the importance of evidence-based practices in music therapy and music-based interventions, it is essential to note that the high number of RCT research in singing intervention studies for respiratory rehabilitation is promising. Most diagnoses included COPD, a progressive condition leading to difficulties in breathing and psychosocial well-being [45]. It is essential to find effective and efficient therapeutic modalities for patients with compromised lung function, and increased RCT studies on singing as a therapeutic and rehabilitative medium are beginning to address this need. By providing scientific results for proposed interventions, singing can be more actively integrated into respiratory rehabilitation.

The results showed that even studies using the same titled program were conducted with different intervention times per session and over different total intervention periods. The diversity of interventions including those provided via home practice is advantageous in terms of flexibility and applicability, especially given environmental constraints and personal preferences due to the COVID-19 pandemic. However, the therapeutic rationale for singing is relatively incomplete and needs to be supplemented through future research.

Given the increasing number of pulmonary patients, current protocols may need to be redesigned to be delivered through multiple channels (in-person and online) and in various contexts (community settings and participants’ homes). New singing protocols should be developed and refined to include virtual or noncontact methodologies. In this review, two studies used only an online method [23,30], and two other studies applied online and in-person treatment components [22,38]. Unlike in recent medical research, there was no clear infection-related trend in the pulmonary rehabilitation research with singing intervention. In music intervention studies, infection issues can arise, as singing in an in-person group or with a music therapist can lend itself to disease transmission [46]. As a result, singing interventions in pulmonary rehabilitation should be reviewed and expanded to use modern technologies such as virtual choirs [47]. 

A noteworthy finding was that the information provided at the pre-singing stage, which included warm-up activities for vocal and physical aspects of singing, was quite specific and highly systematic. This implies that the researchers constructed the pre-singing step through a sufficient understanding of the patient. In pulmonary rehabilitation, pre-singing activities may be critical for preparing patients with pulmonary disease to be aware of their breathing patterns. However, singing activities in the interventions were described in less specific terms and often failed to connect rehabilitation goals with the physical and mental involvement associated with singing. As can be seen from the intervention results, inconsistency in the findings on physical function may be an indication that singing activity should be more tailored to the therapeutic use of music instead of regular singing. Although singing is a familiar musical activity in daily life, the theoretical framework for improving respiratory function should still be used for clinical use.

It is essential to provide the rationale for any intervention. The need for detailed descriptions of the use of music as a therapeutic program and music intervention research procedures was emphasized in previous reviews [48,49]. An intervention should be described at the level suggested in Sheri Robb’s (2011) study to provide more helpful information to readers and for follow-up studies [50]. In future studies, a more significant effect on lung function, the main goal of singing in the studies analyzed here, can be expected through a professional music therapy approach.

Many of the studies in this review included pre-instruction before the session and home practice in their intervention procedures, and previous studies on COPD have emphasized the importance of patient education [51,52]. In our analysis, nearly 2/3 of the studies employed homework to extend the intervention, and prerecorded materials were used to encourage participants to engage in respiratory exercises on a daily basis. These two activities (pre-instruction and home practice) are necessary to improve the patients’ lung function, and this should be reflected in future studies. However, in this review, most studies that included home practice generally failed to present specific guidelines to the participants regarding how to complete their home practice. As a method to increase adherence to an intervention, home practice can be a critical strategy to enhance the effectiveness of singing interventions for patients with pulmonary disease. In future studies, a detailed explanation as to why and how to complete such home practice should be offered.

## 5. Conclusions

Overall, most of the interventions were in the form of a program that contained procedural phases. Most of these interventions were structured around voice production: respiration, vocalization, and singing. With the need for systematic procedures for treating clinical populations, these interventions generally consisted of four phases: physical warm-up, respiration, vocalization, and singing. Of course, the specific activity described in each study varied. While the emphasis on specific musical elements in each phase and detailed activities in each program varied based on the target symptoms, breathing and vocalization were the focus for most of programs.

Singing can be a medium for respiratory exercise in pulmonary rehabilitation. The specific benefits of therapeutic singing varied across the studies. Still, positive effects were evidenced in the patients’ respiration function and psychosocial factors. With the emphasis on self-management in pulmonary rehabilitation, home practice can be a feasible method for teaching patients to manage their pulmonary symptoms. Overall, singing in pulmonary rehabilitation focuses on enhancing pulmonary function. Future studies are needed to identify more efficient and effective intervention procedures. Singing holds great promise in pulmonary rehabilitation, but more research is necessary to ensure that the various needs of patients with pulmonary disease are adequately met.

## Data Availability

Not applicable.

## Appendix A

**Table A1:** Study characteristics.

Author (Year)	Study Design	Diagnosis	Sample Description: Groups: N (Mean Age/Years)	Intervention (Steps, Program)	Measurements
Wade (2002) [28]	Case study time series experimental	Asthma	I: N = 9 (9)	2 steps: (1) Series of vocal exercises; (2) Singing;	PEFR, present mood
Engen (2005) [26]	Case study	COPD Emphysema,	N = 7 (76.34)	5 steps: (1) Relaxation activities; (2) Phonatory exercises; (3) Vocalization; (4) Discussion of music; (5) Singing	FEV, inspiratory threshold (Threshold IMT), 6MWT, breathing (count on visual check/measure by sound level meter), MBS, VAS, the Duke Health Profile
Bonilha et al. (2009) [42]	RCT	COPD	I: N = 15 (69.8) C: N = 15 (73.6)	4 steps: (1) Relaxation exercise; (2) Singing-related respiratory exercise; (3) Vocal exercise; (4) Singing training of Brazilian folk songs	FVC, FEV1, FEV1/FVC, IC, ERV, PImax, PEmax, arterial blood gases while breathing room air, BDI, SGRQ, SaO_2_
Lord et al. (2010) [39]	RCT	COPD	I: N = 15 (66.6) C: 13 (68.1)	4 steps: (Sing for Breathing) (1) Postural work and physical stretches; (2) Breath observation and management; (3) Vocal exercise; (4) Singing songs	HADS, CAT, SF-36, ISWT, BDS, heart rate following the walk, control of breathing (breath hold test, single breath counting)
Irons et al. (2012) [20]	RCT pilot study	Cystic fibrosis	I: N = 14 (7–13), N = 6 (14–17) C: N = 14 (7–13), N = 6 (14–17)	4 steps: (1) Posture; (2) Diaphragmatic breathing; (3) Vocal warmup; (4) Singing	MIP, MEP, FEV1, FVC, FEF25%–75%, CFQ-R
Lord et al. (2012) [40]	RCT	COPD	I: N = 13 (68.8) C (film workshop): N = 11(67.9)	4 steps: (Sing for Breathing) (1) Postural work and physical stretches; (2) Breath observation and management; (3) Vocal exercise; (4) Singing songs	HADS, CAT, SF-36, ISWT, BDS, heart rate following the walk
Goodridge et al. (2013) [18]	RCT feasibility study	COPD	I: N = 14 (70) C (usual care): N = 5 (72)	4 steps: (1) Relaxation and stretching; (2) Singing-related respiratory exercise; (3) Vocalization exercise; (4) Singing songs	6MWT, SGRQ, BIPQ, attendance at the therapeutic singing program and evaluation of adverse, self-reported global health rating, self-reported breathlessness, FEV1
Grasch et al. (2013) [24]	One group pre-post	COPD, emphysema	I: 25 (69.44)	2 steps: (Home practice only) (1) Series of breathing and vocal warmup; (2) Singing songs they choose	Sensormedic 20 C Spirometer, inspiratory & expiratory pressure, SGRQ, Borg scale, BSI-18
Pacheco et al. (2014) [27]	Case study pilot study	COPD	I: 4 (42–68)	3 steps: (1) Relaxation exercises; (2) Vocalization; (3) Singing training for popular Portuguese song	MEP, MIP, 6MWT, SGRQ, CAT, EuroQoL, LCADL, mMRC, FVC, FEV1, FEV1/FVC, TLC, RV
Skingley et al. (2014) [35]	Content analysis	COPD	I: N = 97 (69.5)	2 steps: (1) Relaxation, posture, breathing, and vocal exercise; (2) Singing	SGRQ, MRC breathlessness scale, the YorkSF-12, the EQ5D
Gick & Daugherty (2015) [19]	RCT pilot study	Asthma	I: N = 18 (25.44) C1 (breathing): N = 20 (30.15) C2 (singing): N = 22 (32.77)	2 steps: (1) Diaphragmatic breathing; (2) Singing using a karaoke machine	FEV1, PEFR, MBS, ACQ, SGRQ, Vitality Scale, GHQ, SWLS, PANAS, enjoyment scale
McNaughton et al. (2016) [33]	Grounded theory	COPD, other chronic lung disease	I: N = 12	3 steps: (Sing Your Lungs Out) (1) Warmup and discussion about song repertoire and singing technique; (2) Singing; (3) Warm down	Interview
McNaughton et al. (2017) [25]	One group pre-post pilot study	COPD, other chronic lung disease	I: N = 21 (68.8)	3 steps: (Sing Your Lungs Out) (1) Warmup; (2) Singing; (3) Warm down	6MWT, HADS, CCQ, FEV1, FVC, TLC, RV, FRC, IC
Trivedi (2017) [44]	RCT	COPD	I: N = 26 (NR) C (pulmonary rehabilitation): N = 28 (NR)	2 steps: (1) Pre-singing (respiration, breathing exercise, and demonstration of song and singing technique); (2) Singing	MRC, HADS
Lewis et al. (2018) [31]	Survey evaluation	COPD	I: N = 113 (71.87)	6 steps: (Sing for Lung Health) (1) Physical warmup; (2) Breathing exercise; (3) Voicing; (4) Call and response; (5) Singing with various activities; (6) Cool down	MRC, CAT, PAM, GAD-7, EQ-5D-3L, VAS
Downes et al. (2019) [29]	Program description	COPD	NR NR (NR)	Adaptation of Sing for Lung Health in Uganda 3 steps: (Sing for Breathing Uganda) (1) Physical warmup; (2) Breathing exercise; (3) Singing	The British Lung Foundation SLH evaluation forms
Ganzoni et al. (2020) [41]	RCT single-center randomized, open-label	Cardiomyopathy	I: N = 11 (64) C (standard treatment): N = 11 (65)	Choir singing (HeartChoir)	MIP, MEP, MVO2, MLHFQ
Liu et al. (2019) [43]	RCT	COPD	I: N = 28 (36.85) C (health education): N = 28 (63.3)	4 steps: (Sing for Lung Health) (1) Relaxation exercise; (2) Respiratory exercise; (3) Vocal exercise; (4) Singing exercise	HADS-D, CCQ
Philip et al. (2020) [38]	RCT	COPD	I: N = 9 (72.1) C (usual care): N = 9 (69.89)	5 steps: (Sing for Lung Health) (1) Physical warmup; (2) Breathing exercise; (3) Vocal exercise; (4) Singing; (5) Cool down	SF-36, ABC scale, GAD-7, anxiety, PHQ-9, CAT, MRC, Dyspnea-12, cPPAC, interview
Cahalan, Green, et al. (2022a) [21]	One group pre-post	COPD	I: N = 77 (71.7)	4 steps: (SingStrong) (1) Physical warmup; (2) Breathing; (3) Vocalization exercise; (4) Singing	6MWT, HADS, FEV1, FVC, FEV1/FVC
Cahalan, Meade, et al. (2022b) [22]	One group pre-post	COVID-19, persisting symptoms	I: N = 27 (48.4)	3 steps: (SingStrong) (1) Physical warmup; (2) Vocal exercise and breathing; (3) Singing live and online	C19-YRS, DSQ-SF
Cahalan, Russell, et al. (2022c) [23]	One group pre-post	Pulmonary fibrosis	I: N = 15 (66)	2 steps: (SingStrong for Lung Fibrosis) (1) Vocal exercise and breathing; (2) Singing	SGRQ, IPF-PROM
Clift et al. (2022) [36]	RCT	COPD	I: N = 13 (69.3) C (usual treatment): N = 11 (70)	2 steps: (Singing for Breathing) (1) Breathing exercise; (2) Singing	CAT, mMRC breathlessness scale, SF-36 v2, GAD-7, PHQ-9, FEV1, FVC, 6MWT, SLP
Kaasgaard et al. (2022) [37]	RCT	COPD	I: N = 145 (70) C (PExT): N = 125 (68.8)	4 steps: (Sing for Lung Health) (1) Physical warmup; (2) Vocal warmup with rhythm and pitch games; (3) Singing; (4) Cool down	6MWD, SGRQ, HADS, FEV1, mMRC dyspnea scale, Borg-CR10
Lane et al. (2022) [34]	Phenomenological approach	COPD	Interviewee N = 7 I: N = 13 (69.3) C (usual treatment): N = 11 (70)	2 steps: (Singing for Breathing) (1) Breathing exercise; (2) Singing	Interview based on Clift et al. (2022)
Price & Skingley (2022) [32]	Program evaluation	COPD	I: N = 10 (NR)	(Singing for Better Breathing) Home practice only DVD or YouTube use regularly	A template diary sheet, interview
Smallwood et al. (2022) [30]	Protocol study	COPD, ILD	I: N = 12 (NR)	5 steps: Online (SINFONIA) (1) Check-in; (2) Respiration; (3) Education; (4) Singing; (5) Reflection	SF-36, HADS, Dyspnea-12, CRQ-SAS,6MWT, UCLA-3, patient and carer perceptions of group singing, social dynamics and interactions

Notes. ABC Scale = Activity Balance Confidence Scale; ACQ = Asthma Control Questionnaire; BDI = Basal Dyspnea Index; BDS = Borg Dyspnea Score; BIPQ = Brief Illness Perceptions Questionnaire; Borg-CR10 = Borg Category-Ratio Scale; BSI-18 = Brief Symptom Inventory18; C = control group; CAT = COPD Assessment Test; CFQ-R = Cystic Fibrosis Questionnaire Revised; CCQ = Clinical COPD Questionnaire; COPD = chronic obstructive pulmonary disease; COVID-19 = Corona Virus Disease 19; cPPAC = clinical visit proactive physical activity in COPD; CRQ-SAS = Chronic Respiratory Disease Questionnaire-Self Administered Standardized; C19-YR S = COVID-19 Yorkshire Rehab Screen; DSQ-SF = DePaul Symptom Questionnaire Short Form; EQ5D = European Quality of Life 5 Dimensions; EQ-5D-3L = European Quality of Life 5 Dimensions 3 Level Version; ERV = expiratory reserve volume; FEF25%-75% = forced expiratory flow 25% to 75%; FEV1/FVC = forced expiratory volume at 1 s/forced vital capacity; FEV = forced expiratory volume; FEV1 = forced expiratory volume in 1 s; FRC = functional residual capacity; FVC = forced vital capacity; GAD-7 = General Anxiety Disorder–7; GHQ = General Health Questionnaire; HADS = Hospital Anxiety and Depression Scale; HADS-D = Hospital Anxiety and Depression Scale Depression Subscale; I = intervention group; ILD = interstitial lung disease; IC = inspiratory capacity; IPF-PROM = idiopathic pulmonary fibrosis-patient reported outcome measures; ISWT = Incremental Shuttle Walk Test; LCADL = London Chest Activity of Daily Living Scale; MBS = Modified Borg Scale; MEP = maximal expiratory pressure; MIP = maximal inspiratory pressure; MLHFQ = Minnesota Living with Heart Failure Questionnaire; mMRC = Modified Medical Research Council Dyspnea Scale; MRC Dyspnea Scale = Medical Research Council Dyspnea Scale; MVO2 = myocardial volume oxygen; N = number; PAM = Patient Activation Measure; PANAS = Positive And Negative Affect Scale; PEFR = peak expiratory flow rate; PEmax = maximal expiratory pressures at the mouth level; PImax = maximal inspiratory pressure at the mouth level; PHQ-9 = Patient Health Questionnaire; QoL = quality of life; RCT = randomized controlled trial; RV = residual volume; SaO_2_ = measurement of arterial oxygen saturation; SF-36 = Short Form 36; SF-36 v2 = Short Form 36 version 2; SGRQ = St. George’s Respiratory Questionnaire; SLP = structured light plethysmography; SWLS = Satisfaction With Life Scale; Threshold IMT = threshold inspiratory muscle training; TLC = total lung capacity; UCLA-3 = University of California Los Angeles Loneliness Scale Version 3; VAS = Visual Analogue Scale; YorkSF-12 = York Short Form-12; 6MWD = 6 Minute Walk Distance Test; 6MWT = 6 Minute Walking Test.
